# Clinical Outcomes of Extreme Lateral Interbody Fusion in the Treatment of Adult Degenerative Scoliosis

**DOI:** 10.1100/2012/680643

**Published:** 2012-09-24

**Authors:** Adam M. Caputo, Keith W. Michael, Todd M. Chapman, Gene M. Massey, Cameron R. Howes, Robert E. Isaacs, Christopher R. Brown

**Affiliations:** ^1^Department of Orthopaedic Surgery, Duke University Medical Center, P.O. Box 2807, 335 Baker House, 200 Trent Drive, Durham, NC 27710, USA; ^2^Division of Neurosurgery, Department of Surgery, Duke University Medical Center, Durham, NC 27710, USA

## Abstract

*Introduction*. The use of extreme lateral interbody fusion (XLIF) and other lateral access surgery is rapidly increasing in popularity. However, limited data is available regarding its use in scoliosis surgery. The objective of this study was to evaluate the clinical outcomes of adults with degenerative lumbar scoliosis treated with XLIF. *Methods*. Thirty consecutive patients with adult degenerative scoliosis treated by a single surgeon at a major academic institution were followed for an average of 14.3 months. Interbody fusion was completed using the XLIF technique with supplemental posterior instrumentation. Validated clinical outcome scores were obtained on patients preoperatively and at most recent follow-up. Complications were recorded. *Results*. The study group demonstrated improvement in multiple clinical outcome scores. Oswestry Disability Index scores improved from 24.8 to 19.0 (*P* < 0.001). Short Form-12 scores improved, although the change was not significant. Visual analog scores for back pain decreased from 6.8 to 4.6 (*P* < 0.001) while scores for leg pain decreased from 5.4 to 2.8 (*P* < 0.001). A total of six minor complications (20%) were recorded, and two patients (6.7%) required additional surgery. *Conclusions*. Based on the significant improvement in validated clinical outcome scores, XLIF is effective in the treatment of adult degenerative scoliosis.

## 1. Introduction

Adult degenerative scoliosis has an estimated prevalence of 6% in people over the age of 50 [1]. Patients classically present with back pain, sagittal imbalance, or radicular symptoms. Though conservative management is recommended as an initial treatment, outcomes are frequently unacceptable [2].

When nonoperative treatment fails, adult degenerative scoliosis presents significant surgical challenges. Decompression may be the treatment of choice in mild deformity or minimal instability. However, decompression alone has been associated with a risk of iatrogenic instability and progression of deformity [3, 4]. For this reason, an instrumented arthrodesis is often indicated [4–7].

Interbody fusion has been demonstrated to be an effective method of deformity correction in adult scoliosis [8, 9]. Approaches to interbody fusion include posterior lumbar interbody fusion (PLIF), transforaminal lumbar interbody fusion (TLIF), and anterior lumbar interbody fusion (ALIF). In 2006, a lateral transpsoas approach to the lumbar spine was described [10]. This approach has been popularized as “extreme lateral interbody fusion (XLIF).” Advantages of the lateral approach may include decreased blood loss, accelerated recovery, and decreased cost [11–13].

Recently, authors have described the use of lateral interbody fusion for the treatment of a variety of lumbar conditions [14–23]. The indications for lateral access surgery continue to expand as more surgeons adopt the technique. However, with this increase in popularity comes a need for more clinical data. In an attempt to address this need, this study describes a single surgeon's experience with XLIF in the treatment of adult degenerative scoliosis.

## 2. Methods

### 2.1. Study Design

The study herein was an institutional review board-approved evaluation of adult degenerative scoliosis treated by a single surgeon at a major academic institution. During the study period, thirty consecutive patients underwent XLIF with supplemental posterior instrumentation. Validated clinical outcome scores were obtained preoperatively and at most recent followup for comparison purposes. Complications were recorded.

### 2.2. Subjects

Thirty patients were followed up for an average of 14.3 months (Table 1). Inclusion criteria required a diagnosis of symptomatic degenerative adult scoliosis that had failed at least a year of conservative treatment. Patients were required to have a coronal Cobb angle of at least 10° for inclusion. The average age was 65.9 years (range 53–76 years). The study included 11 men and 19 women with an average BMI of 28.8 (range 19–38). 18 patients had apex-left deformity, and 12 had apex-right. Nine patients were active smokers at the time of surgery. 15 patients (50%) had undergone prior lumbar spine surgery at one or more levels: 6/30 laminectomy, 1/30 interspinous spacer placement, 1/30 microdiscectomy, 1/30 anterior/posterior fusion, and 6/30 posterolateral fusion.

### 2.3. Surgical Technique

**Figure 1:**
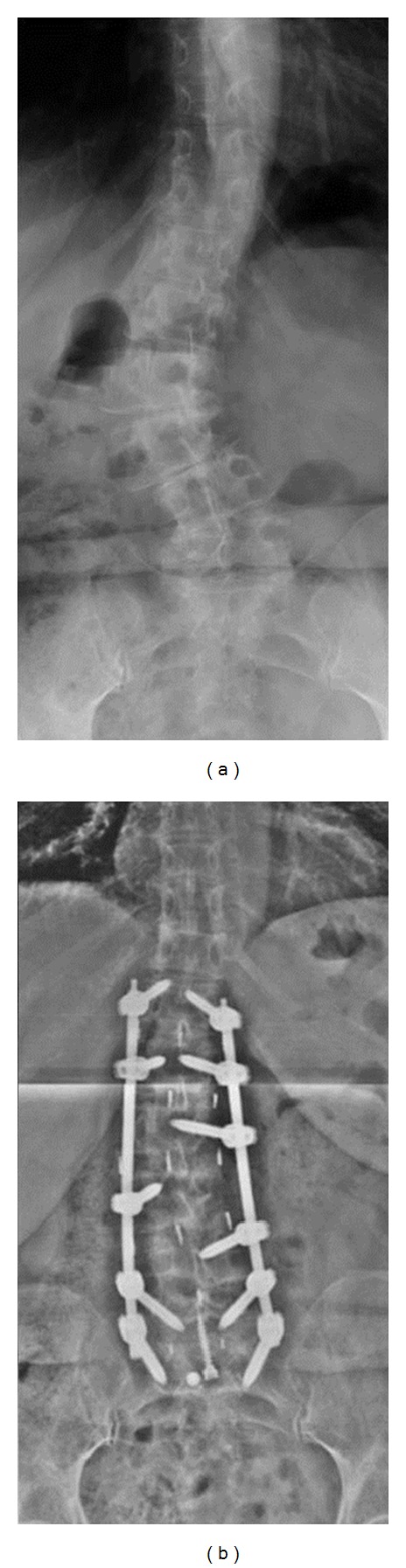
Preoperative (a) and postoperative (b) anteroposterior radiographs of the lumbar spine in a patient treated with XLIF with percutaneous pedicle screws and rods.

Interbody fusion was completed using the XLIF technique (NuVasive, Inc., San Diego, CA) as described by Ozgur et al. [10]. Laterally placed interbody spacers were supplemented with Osteocel Plus allograft cellular bone matrix (NuVasive, Inc., San Diego, CA). Lateral approaches were made from the concave side. Posterior instrumentation involved percutaneous placement of transpedicular screws and rods (SpheRx, DBR, NuVasive, Inc., San Diego, CA) (Figure 1). A total of 127 levels from T10 to L5 (average of 4.2 levels; range 1–7 levels) were treated using XLIF. In addition to XLIF, traditional anterior interbody fusion (ALIF) was used in 11 patients who required an L5-S1 fusion. Typically, all required procedures were performed during a single operative session. However, in patients requiring ALIF, the ALIF and instrumentation portions of the case were performed two days after the XLIF portion.

### 2.4. Clinical Outcome Scores

Validated clinical outcome scores were obtained on all patients preoperatively and at most recent followup. Outcome scores included the Oswestry Disability Index (ODI), short form-12 (SF-12) and visual analog pain score (VAS) for back and leg pain. Complications were recorded as any deviation from a normal postoperative course.

### 2.5. Statistical Analysis

Frequency statistics were used to characterize patient demographics and treatment variables. Clinical outcome scores were evaluated with paired *t*-tests using SPSS v. 19.0 (SPSS IBM, Inc. Chicago, IL). Statistical significance was defined as *P* < 0.05.

## 3. Results

### 3.1. Clinical Outcome Scores

The study group demonstrated a significant improvement in multiple clinical outcomes scores from preoperative to most recent followup (Table 2). The average ODI decreased from 24.8 to 19.0, a significant improvement (*P* < 0.001). The average SF-12 mental and physical component scores improved, although the change was not significant. The average VAS back pain score decreased from 6.8 to 4.6, a significant improvement (*P* < 0.001). The average VAS leg pain score decreased from 5.4 to 2.8, a significant improvement (*P* < 0.001).

### 3.2. Postoperative Complications

Of the thirty patients who underwent surgery, eight (26.6%) were noted to experience complications. Two patients had lateral wound breakdown which was healed via secondary intention. One patient had a pedicle fracture at T12. This patient was asymptomatic and did not require additional intervention. One patient developed a symptomatic nonunion at L1-L2. This patient returned to the operating room 13 months after his initial procedure for revision fusion and extension of hardware. One patient developed a hernia at his lateral incision and underwent an elective hernia repair by a general surgeon several months after his initial procedure. One patient had uncontrolled atrial fibrillation after the XLIF stage of her reconstruction. As a result, the posterior instrumentation stage was delayed until six weeks after the XLIF stage.

Two patients had iatrogenic rupture of the anterior longitudinal ligament (ALL). Rupture of the ALL is often considered a technical deviation during XLIF. However, for the purposes of this paper, it was recorded as a complication. In one of the patients, the ALL rupture occurred at L4-L5. To address this, an anterior plate was placed across L4-5 during the planned ALIF portion of the case. In the second patient, the ALL rupture was at L3-L4. To provide additional stability, a lateral plate was placed during the XLIF exposure. Both of these patients went on to an uncomplicated fusion, which was confirmed with thin-cut computed tomography one year after surgery.

It is also notable that a substantial portion of patients reported anterior thigh pain/numbness after surgery. However, the authors did not consider this a “complication” given that it is expected to occur in a sizable percentage of patients undergoing the transpsoas approach. If the patient's symptoms persisted beyond the immediate postoperative period, it was recorded as a complication. However, in the studied population, all reported anterior thigh pain and numbness had resolved by 4 weeks.

## 4. Discussion

Historically, scoliosis correction has involved a combined anterior/posterior approach or a posterior-only approach. Though these techniques have been demonstrated to improve clinical outcomes, they are also associated with a high complication rate [24, 25]. Specifically, the anterior approach is associated with bowel injuries, ileus, vascular injury, and retrograde ejaculation [26–28]. Posterior approaches necessitate exposure of the dura and nerve roots, placing them at greater risk for injury. A recent large study by Pateder et al. [29] revealed a complication rate of up to 45% for traditional scoliosis surgery.

Recent studies have indicated that surgical morbidity may be reduced with the use of less invasive techniques such as XLIF. A multicenter study by Isaacs et al. [11] involving a separate patient population more than the study herein demonstrated that the perioperative morbidity of XLIF in the treatment of adult degenerative scoliosis compares favorably to more invasive techniques. A study by Youssef et al. [12] demonstrated fewer complications and quicker recoveries in patients with lumbar degenerative disease treated with XLIF.

Besides decreased morbidity, there may also be biological benefits to XLIF and other lateral access surgery. With ALIF, PLIF, or TLIF, there is a mandatory breach of both the annulus and a longitudinal ligament. However, XLIF allows for preservation of the anterior and posterior longitudinal ligaments, conserving stability at treated levels. Additionally, XLIF allows for placement of a wide cage that rests on strong peripheral bone, potentially reducing the risk of cage subsidence [30].

Due to these potential benefits, lateral access surgery has rapidly increased in popularity. As more surgeons adopt the technique, the indications for lateral access surgery have broadened to include scoliosis surgery. However, more data is needed regarding the clinical outcomes of scoliosis patients treated with XLIF and other lateral access surgery. Specifically, there are few studies in the literature looking at the clinical outcomes of patients with adult degenerative scoliosis treated with lateral access techniques [30–35].

The study herein reports a single surgeon's experience with thirty consecutive patients treated with XLIF. A comprehensive panel of validated outcome measurements (ODI, VAS pain scores, and SF-12) were used to evaluate outcomes and clinical efficacy. Surgery led to improvement in multiple parameters, including a statistically significant improvement in ODI, VAS back pain, and VAS leg pain scores. The improvement in these outcomes supports the efficacy of XLIF in the treatment of adult degenerative scoliosis and adds to a growing body of literature supporting the effectiveness of XLIF in the treatment of scoliosis surgery. Furthermore, despite the advanced age of this study population (average age 65.9 years), the complication rate was low (26.6%) when compared to traditional approaches [29]. With the exception of two patients requiring additional surgery (one revision fusion, one elective hernia repair), the complications were minor and resolved without further intervention. In comparison, a recent study by Daubs et al. [24] using traditional approaches with a comparable population (average age 67 years) reported a complication rate of 37% with a major complication rate of 20%.

This study had several notable limitations. The most significant is a lack of a comparison group of conventional posterior or anterior/posterior approach patients. In addition, the followup period is modest, and long-term studies are needed. Nevertheless, this study supports the efficacy of XLIF in the treatment of adult degenerative scoliosis using a panel of validated clinical outcome scores with relatively large series of patients.

## 5. Conclusions

The goal of this study was to evaluate the role of XLIF in the treatment of adult degenerative scoliosis. In a series of thirty patients, significant clinical improvement was noted in multiple-validated outcome measurements. This series adds to a growing body of data supporting the efficacy of XLIF in the treatment of adult degenerative scoliosis. Though not without complications, XLIF was associated with less major complications and a lower overall complication rate than traditional approaches. In order to further clarify the role of XLIF in scoliosis surgery, long-term and comparative studies are needed.

## Figures and Tables

**Table 1:** Characteristics of thirty patients treated with XLIF.

Age (years)	65.9 (53–76)
Sex	11 men; 19 women
BMI	28.8 (19–38)
Deformity	18 apex-left; 12 apex-right
Cobb Angle	20.2° (10.1°–42.0°)

**Table 2:** Clinical outcomes in thirty patients treated with XLIF.

	Preoperative	Postoperative	*P* value
ODI	24.8	19.0	<0.001*
SF-12M	62.8	64.2	0.20
SF-12P	28.6	32.3	0.07
VAS Back	6.8	4.6	<0.001*
VAS Leg	5.4	2.8	<0.001*

*Statistically significant.
